# Development and Evaluation of Relational Leadership Guidelines for Primary Healthcare: A Mixed Methods Study

**DOI:** 10.1155/jonm/2653041

**Published:** 2025-10-29

**Authors:** Lillian Kalimashe, Emmerentia du Plessis, Leepile Alfred Sehularo

**Affiliations:** ^1^Department of Health, West Rand District Health Services, Gauteng Department of Health, Johannesburg, South Africa; ^2^NuMIQ Research Focus Area, Faculty of Health Sciences, North-West University, Potchefstroom, South Africa; ^3^Lifestyle Diseases Research Entity, Faculty of Health Sciences, North-West University, Mafikeng, South Africa

**Keywords:** guidelines, innovation, mixed-methods, primary healthcare, relational leadership, shared decision-making

## Abstract

**Background:**

Primary healthcare (PHC) settings are facing unprecedented challenges in the wake of rapid technological, demographic, and systemic changes. Traditional hierarchical models, with their rigid structures and top–down decision-making, have increasingly proven inadequate in addressing the complex needs of modern healthcare. In contrast, relational leadership (RL) emphasizes emotional intelligence, collaboration, inclusivity, and shared decision-making thus offering a promising alternative. This study seeks to develop and evaluate practical guidelines for employing RL to enhance adaptability, flexibility, and innovation in PHC environments.

**Methods:**

A descriptive, exploratory concurrent parallel mixed-method design was utilized to capture the multidimensional aspects of leadership in PHC. Quantitatively, the Relational Leadership Questionnaire (RLQ) was administered to assess prevailing leadership practices and perceptions within various healthcare settings. Qualitatively, action learning methods, including reflective journals, group sessions, and observational notes, were employed to gather in-depth insights into the real-world dynamics and challenges encountered by healthcare leaders. This comprehensive approach ensured that the developed guidelines were both empirically robust and contextually relevant.

**Results:**

The findings demonstrate that RL significantly strengthens team cohesion, stakeholder collaboration, and overall organizational performance within PHC. Core elements such as enhanced emotional intelligence, shared decision-making, and the strategic leveraging of social and human capital were found to mitigate challenges like role overload, resource constraints, and entrenched hierarchical barriers. Importantly, the study identifies targeted training and structural adjustments as critical means to facilitate a smooth transition from traditional leadership models to more adaptive, relational approaches.

**Conclusion:**

The evaluated guidelines present a transformative framework for PHC leadership. By shifting from hierarchical to RL, healthcare organizations can foster environments that are more inclusive, innovative, and responsive to both clinical and administrative demands. These findings lay the groundwork for ongoing leadership development initiatives, and future research aimed at reinforcing adaptive practices in diverse healthcare contexts.

## 1. Introduction

The healthcare sector is undergoing overwhelming changes, driven by rapid advancements in medical technology, changing patient demographics, and the increasing complexity of healthcare delivery systems [1]. Within this dynamic environment, effective leadership has become key. According to a study conducted by Endalamaw et al., traditional hierarchical leadership models, which depend on rigid structures and top–down decision-making, have shown their limitations in addressing the complexities of modern healthcare systems [2]. This challenge is even more evident in crisis-prone PHC, where adaptability, flexibility, and innovation are vital for responding to the evolving needs of patients and communities [3].

RL has emerged as a potential alternative to conventional leadership paradigms. Unlike traditional hierarchical approaches that prioritize unidirectional authority, RL is basically participatory, fostering interconnectedness among healthcare teams to enhance both individual and organizational performance [4, 5]. Relational leadership theory (RLT) conceptualizes leadership not as a characteristic or behavior of individual leaders but as a process of social influence and a concept that aligns strongly with complexity leadership theory, occurring through relationships [6]. Building these relationships involves the creation of strong networks within and between healthcare teams, fostering a sense of community and shared accountability that supports effective collaboration [7]. Distributed decision-making empowers team members at different levels, encouraging ownership of outcomes and increasing organizational efficiency [8]. By prioritizing the social processes of leadership, nurturing environments that support team cohesion, emphasizing collaboration, inclusivity, mutual respect, and ethical practices, RL aims to strengthen connections between leaders and their teams [5, 9, 10]. Sun believes that these strengthened relationships not only improve organizational effectiveness but also encourage a culture of innovation and adaptability, making RL particularly appropriate for PHC environments [11].

According to a study conducted in South Africa [12], PHC systems often face problems such as resource constraints, diverse patient needs, and complicated care delivery challenges. Other longstanding PHC challenges are fragmented service delivery, inequitable resource distribution, and reduced team cohesion [5, 13]. It is for such reasons these settings often require leadership frameworks adaptable enough to navigate interprofessional collaboration, patient-centered care, and system-level integration [14, 15]. The RL approach aligns particularly well with PHC settings for several reasons. First, PHC delivery increasingly depends on interdisciplinary teams including physicians, nurses, allied health professionals, and administrative staff, needing leadership that facilitates effective collaboration. Second, the patient-centered care model central to PHC requires leadership that appreciates diverse perspectives, including those of team members, patients, and community members. Third, PHC's emphasis on prevention and health promotion demands leadership practices that can build trust and sustain engagement across numerous stakeholders. RL, with its focus on emotional intelligence, fostering resilience, team member empowerment, ethical leadership practices, and shared decision-making, offers a valuable framework to address these complexities while encouraging positive organizational culture [8, 16].

Despite its theoretical appeal and alignment with latest healthcare principles, the practical integration of RL remains inconsistent across settings. Some studies have demonstrated its correlation with positive outcomes, such as reduced turnover, enhanced employee engagement, and improved patient safety [17, 18]. However, methodological weaknesses also persist in the existing body of literature. For instance, while quantitative surveys effectively quantify leadership's impact on metrics such as job satisfaction or mortality rates [19], they often disregard the subjective dimensions critical to relational dynamics, such as trust-building and conflict resolution [20]. Similarly, qualitative explorations tend to lack standardized replicability, presenting rich but split narratives [21], underscoring the need for integrated methodological approaches. While the District Innovation and Action Learning for Health Systems Development (DIALHS) collaboration has successfully operationalized RL through action learning groups to strengthen PHC leadership [5], repeating these outcomes in diverse healthcare environments remains difficult [22], particularly where resource limitations and increased workload are coupled with rooted hierarchical systems [23].

Given the importance of clarifying the dimensions and characteristics of RL in strengthening leadership practices within PHC, researchers can assess the effectiveness of leadership behaviors and team dynamics through the application of RL principles. Additionally, PHC settings, managers, and policymakers can use the insights from these guidelines to enhance the quality and responsiveness of leadership in PHC settings. With this background in mind, the present study aimed to develop and evaluate comprehensive evidence-based guidelines for RL in PHC, South Africa.

## 2. Methods

### 2.1. Study Design

This study employed a descriptive, exploratory concurrent parallel mixed-method design, meaning quantitative and qualitative data were collected and analyzed at the same time but separately, and integration only done with the findings from both data. The study was carried out over a period of 6 months (April to September 2024). Across the West Rand district health, there are 47 PHC facilities which provide PHC services to their population. The study used the Relational Leadership Questionnaire (RLQ) for quantitative assessment and action learning methodologies for qualitative exploration. The RLQ provided a structured framework for measuring RL competencies, while action learning facilitated iterative and collaborative problem-solving, enabling leaders to adapt to their dynamic healthcare environments [24]. Further elaboration of the quantitative and qualitative findings from each study subphase is available upon request as part of the larger research initiative informing this guideline development. By combining RLQ assessment with action learning, this study adopted a comprehensive approach to understanding and improving RL in PHC settings.

**Figure 1:**
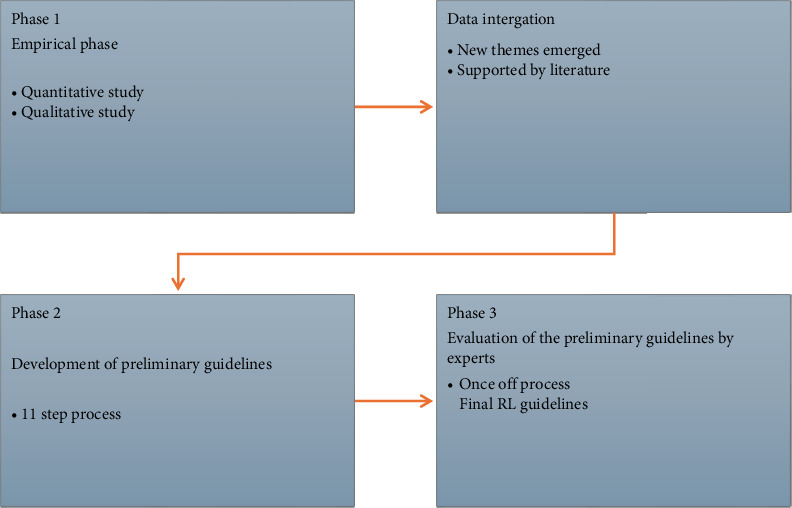
Mixed-method design flowchart—Phase 1–3.

The study was carried out in 3 phases (Figure 1) namely, phase 1: empirical phase (quantitative study—phase 1: subphase 1; qualitative study—phase 1: subphase 2). The quantitative and qualitative subphases' data collection was done at the same time, over a period of 6 months (April–September 2024), analyzed separately and only integrating the findings from both; phase 2: data integration and development of guidelines; and phase 3: evaluation of the preliminary guidelines and reporting on the final guidelines. See steps followed for the study in Figure 1 below.

### 2.2. Phase 1: Subphase 1

#### 2.2.1. Quantitative Study

##### 2.2.1.1. Study Design

This quantitative phase was conducted as a component of the descriptive, exploratory concurrent parallel mixed-method design used in the study to examine RL in PHC systems. The research employed a descriptive cross-sectional design using a structured RLQ. The RLQ was administered to PHC personnel within a health district with four subdistricts in the Gauteng Province, South Africa.

##### 2.2.1.2. Sample

The sample size for the quantitative study was calculated using the formula used in a previous study by Carifio (*β* = 0.2, *α* = 0.05, correlation coefficient: *r* = 0.27) [25]. The sample size was estimated at 300 employees. Three hundred and seventeen participants filled out the questionnaires. The inclusion criteria considered in this study were being willing to participate in the study, having at least 1 year of permanent work experience in PHC, and reporting to a supervisor in an official leadership position. The exclusion criteria were students allocated within the district's facilities for work integrated learning, employees working on temporary contractual basis, and those unwilling to participate in the study for any reason. This guaranteed that the study includes employees with extensive experience and understanding of their supervisors' leadership styles, delivering accurate data on RL perceptions.

##### 2.2.1.3. Data Collection Method

Quantitative data were collected using structured RLQ surveys, measuring RL subscales such as inclusivity, empowerment, caring, ethics, and vision. Surveys were disseminated both digitally and in print to maximize responses and allow those without access to digital or online platforms to participate. Potential participants were also provided with informed consent letters to sign prior completing the survey.

##### 2.2.1.4. Data Collection Instrument

Data were collected using a demographic survey, and the RLQ was the instrument used to collect the data in this study. The *demographic checklist* included questions on age, gender, number of years permanently employed in the health district, job title, and specific subdistrict they work in. The designed and validated RLQ [25] contains 25 close-ended statements and measures five subscales: inclusiveness (5 questions), empowerment (5 questions), caring (5 questions), ethicality (5 questions), and vision (5 questions); the items are scored using a 7-point Likert scale ranging from 1 “*strongly disagree*” to 7 “*strongly agree*” in a self-report manner. The minimum score of this questionnaire is 25, and its maximum score is 175. The original developer of the RLQ did not provide conventional cut-off scores for understanding RL levels [25]; hence, a categorization framework was created to facilitate meaningful analysis and interpretation. For this study, the researchers put the scores into three groups based on equal intervals: low RL (25–74), medium RL (75–124), and high RL (125–175). The subscale' scores 5–14 indicate low score; 15–24 indicate medium score; and 25–35 indicate high score per subscale. This method aligns with psychometric standards in the absence of established thresholds and is endorsed by contemporary research promoting context-sensitive and theoretically informed modifications in RL measurement [26]. The classification represents the complete distribution of scores identified in the study population and corresponds with the RLQ's theoretical framework, which evaluates five fundamental RL traits.

##### 2.2.1.5. Data Analysis

Statistical analysis was performed using SPSS Version 28. Descriptive statistics (means, medians, and standard deviations), *t*-tests, chi-square tests, Pearson's correlation coefficients, and multiple regression analyses were applied to determine the extent to which employees perceived their leaders to be practicing RL. The total scores for RL as well as the scores for each subscale are shown as mean and standard deviation.

Based on participant responses, the mean total RLQ score was 71.64 out of 175, indicating that 60.5% of employees perceived low RL in their settings. Subscale distributions further revealed that vision (76.4%) and ethicality (66.5%) were the lowest-rated domains, while empowerment (44% low, 26.2% high) showed moderate variability. Inclusivity and caring were also scored low by 60.9% and 53.6% employees, respectively.

##### 2.2.1.6. Establishing Rigor

Reliability was ensured through Cronbach's alpha analysis to validate internal consistency, while the all-inclusive sampling minimized selection bias. RLQ internal consistency and dependability are high, with an alpha Cronbach value of 0.90 in its initial validation and 0.96 in this study [25]. The RLQ's reliability assures that employees' RL perceptions are accurately reflected.

### 2.3. Phase 1: Subphase 2

#### 2.3.1. Qualitative Study

##### 2.3.1.1. Study Design

The qualitative component of the study was conducted using an action learning approach, which included group sessions, reflective journal entries, and researcher observational notes. This subphase aimed to explore the lived experiences, perceptions, and behavioral practices associated with RL among PHC leaders. The action learning design was chosen for its effectiveness in fostering reflective practice and collaborative learning among leaders. This approach emphasizes solving real-world organizational challenges by encouraging participants to reflect on problems, share insights, and work together on practical solutions [13]. The action learning approach commenced with a workshop to introduce and enhance participants' knowledge of the RL concept. The workshop was detailed such that healthcare leaders could critically evaluate their leadership approaches relating to the principles of RL [14].

##### 2.3.1.2. Sample

The 10 participants were purposively selected for maximum variation in professional experience, leadership roles, and facility contexts. Eligibility criteria included a willingness to engage in structured action learning group sessions, and at least 6 months of leadership experience in the PHC setting. The sample comprised clinical managers, administrative leaders, and front-line supervisors with a wide variety of leadership expertise.

##### 2.3.1.3. Data Collection

Data collection involved various methods designed to capture comprehensive insights into RL dynamics. Action learning group sessions were facilitated by the principal researcher and recorded with participant consent, allowing leaders to collaboratively explore strategies for implementing RL principles within their respective contexts. The data were transcribed verbatim on the day of each session. Reflective journals enabled participants to systematically document their leadership experiences, challenges, and decision-making processes, offering nuanced perspectives on everyday leadership practices. Additionally, researcher observations were conducted to record nonverbal interactions and real-time leadership dynamics, contributing to a richer understanding of RL in practice. Participants were informed of the objectives of the research, assured of confidentiality, and provided written consent. Participation was entirely voluntary, and all participants were offered the opportunity to clarify or elaborate on their contributions at the end of each session.

##### 2.3.1.4. Data Analysis

Data were analyzed by the first author and co-coder using Creswell's thematic analysis framework [27], incorporating literature control to contextualize themes. Codes were generated inductively from the data and clustered into categories based on similarity. A total of 5 primary themes and 22 subthemes were identified across the reflective journals, session transcripts, and primary researcher's observational data. Key themes that emerged were “understanding and implementation of RL,” “benefits of RL,” “challenges while implementing RL,” “connection between RL and patient care” alongside “strategies to enhance the practice and implementation of RL.” These qualitative insights added essential context and depth to the quantitative findings, directly informing the structure and content of the RL guidelines by illustrating the practical barriers and facilitators present in PHC leadership.

##### 2.3.1.5. Establishing Rigor

Trustworthiness of the qualitative findings was ensured through multiple strategies. Credibility was achieved by employing triangulation, which involved integrating stakeholder perspectives, researcher observations, and literature-based insights to provide a robust and multifaceted understanding of RL practices. Additionally, member checking was conducted by sharing interpretations with PHC leaders who participated in the action learning sessions, enabling them to validate or refine the researcher's understanding of their perspectives.

To strengthen transferability, the study provided thick descriptions of the PHC context, participant demographics, and leadership structures, allowing readers to evaluate the relevance and applicability of the findings to similar healthcare settings. Dependability was maintained by documenting the research process through an audit trail, which included detailed notes on data collection, analysis decisions, and theme development, thus ensuring that the study's methodological choices were transparent and replicable. Finally, confirmability was supported using reflexive journaling, where the researcher critically reflected on personal biases and assumptions throughout the research process to ensure that findings emerged from participants' data rather than researcher preconceptions.

### 2.4. Integration and Development of Preliminary Guidelines (Phase 2)

The development of preliminary guidelines for RL in PHC was grounded in a systematic integration of quantitative and qualitative findings from Phase 1. Quantitative data from the RLQ revealed significant gaps in leadership practices, with notably low scores in inclusivity (mean: 13.96/35), vision (76.4% of staff rating leaders as low), and ethical leadership (66.5% low scores). Qualitative insights from action learning sessions and reflective journals further contextualized these findings, highlighting challenges such as unilateral decision-making, difficulties in balancing accountability with empathy, and insufficient communication of organizational vision.

**Figure 2:**
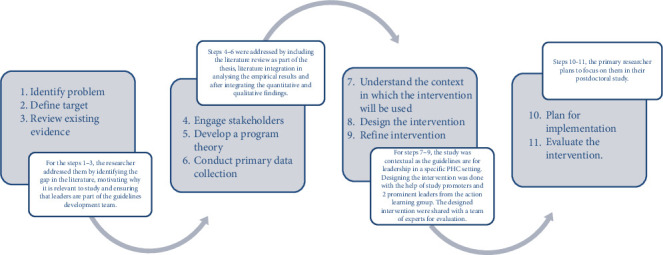
11-step process for guidelines development when considering complexity [28] application to this study.

The new themes from the integrated mixed-method findings were used to develop guidelines following O'Cathain et al.'s 11-step guideline development framework thus ensuring methodological rigor [28]. The framework emphasizes iterative, stakeholder-driven processes grounded in evidence and context (see Figure 2 for the representation of the framework). The process began with problem identification, mapping leadership deficits against empirical data. The target audience being PHC leaders was defined early to ensure relevance. A comprehensive literature review supported the evidence base, while stakeholder engagement throughout ensured practical alignment. A program theory was articulated to link RL behaviors to improved team and patient outcomes. Primary data collection through mixed methods provided contextual depth, and the implementation setting was analyzed to tailor the guidelines to PHC realities. The guidelines were then designed and refined through expert feedback, and their development was documented transparently. From this development process, eight core RL domains emerged, each supported by subthemes and actionable recommendations. For instance, the theme “Fostering Inclusivity and Participation” included strategies such as building psychological safety and equitable decision-making, directly addressing the low inclusivity scores identified in the RLQ. Similarly, “Enhancing Empowerment Through Mentorship and Delegation” responded to qualitative findings that revealed staff desire for greater autonomy and professional growth opportunities.

Although implementation and evaluation steps are planned for future research, the framework ensured that the guidelines were both methodologically robust and practically grounded in the lived experiences of PHC leaders. The preliminary guidelines were structured as clear, actionable statements to facilitate implementation. For example, to “Uphold Ethical Leadership and Transparent Decision-Making,” leaders were advised to “model accountability and humility by taking responsibility for failures and encouraging team members to voice concerns without fear of retribution.” This direct linkage between empirical findings and practical recommendations ensured the guidelines were both evidence-based and feasible for PHC settings.

### 2.5. Expert Evaluation and Finalization of Guidelines (Phase 3)

The preliminary guidelines underwent rigorous evaluation by an expert panel to assess their validity, clarity, and applicability. The panel comprised 10 specialists with expertise in healthcare leadership, relational care, and guideline development, ensuring a comprehensive review (see Table 1 for the attributes of experts). Using a once-off evaluation method, experts rated the guidelines on a 3-point scale across six criteria: clarity, comprehensiveness, credibility, applicability, adaptability, and validity.

The evaluation revealed strong consensus on the guidelines' robustness, with particularly high scores for comprehensiveness (3/3) and credibility (2.9/3). The experts praised the alignment of the guidelines with broader principles such as Ubuntu (emphasizing interconnectedness) and their focus on patient-centered care. However, minor refinements were suggested to enhance clarity, including the use of bullet points for readability and the incorporation of active verbs in thematic titles (e.g., changing “Foster Inclusivity” to “Foster inclusivity by building diverse relationships”). Additionally, experts recommended strengthening strategies for role modeling and patient feedback integration to ensure sustained practice.

The finalized guidelines incorporated these refinements, resulting in a more streamlined and actionable framework. For example, the theme “Integrate Patient-Centered Leadership Practices” was expanded to include specific metrics for tracking patient satisfaction and regular community feedback sessions. This iterative refinement process underscored the importance of expert evaluation in ensuring that guidelines are not only theoretically sound but also practically implementable in diverse PHC contexts.

### 2.6. Findings

Participants in the empirical phase included PHC personnel and leaders such as facility managers and coordinators. The study recruited a diverse group of participants representing various professional backgrounds, with work experience ranging from novice to experienced leaders. This ensured that the insights collected reflected a broad spectrum of perspectives on leadership practices in PHC settings.

The research employed a mixed-methods approach, integrating quantitative and qualitative subphases. The quantitative phase involved structured assessments using the RLQ to evaluate leadership practices. Meanwhile, the qualitative subphase included action learning sessions, reflective journal entries, and researcher observational notes, allowing for an in-depth exploration of RL experiences. Upon completion of the data analysis, the study identified 5 primary themes and 22 subthemes from the qualitative subphase, while statistical trends from the quantitative subphase highlighted several RL gaps. Specific gaps noted included low inclusivity scores, moderate empowerment scores, and deficiencies in visionary leadership, caring, and ethical leadership.

From the integration of these findings, eight core themes emerged as critical to RL practices in PHC. These themes encompass fostering inclusivity and participation, enhancing empowerment through mentorship, developing and communicating a shared vision, exhibiting caring and empathy, upholding ethical leadership, promoting continuous learning, integrating patient-centered leadership, and employing context-based leadership with feedback. Each theme was validated through an extensive literature review, ensuring alignment with existing studies on RL effectiveness in healthcare.

To translate these findings into guidelines with actionable strategies, the study applied a structured 11-step guideline development framework for complex interventions [28]. The framework was used to ensure methodological transparency and contextual dependability. The structured inclusion of stakeholder voices throughout the research added credibility, while the expert review ensured practical alignment with everyday realities in PHC. The 10 experts affirmed that the final guidelines were clear, comprehensive, and adaptable, with an average evaluation score of ≥ 2.9 across six core criteria. However, feedback also noted areas for further refinement, including simplified language and flexible implementation strategies tailored to resource-constrained environments.

The final practice guidelines for RL in PHC were presented using the AGREE Reporting Checklist to ensure quality and clarity. They aim to promote inclusivity, empowerment, ethical practice, and patient-centered care among healthcare leaders and teams. The guidelines target leaders, managers, and multidisciplinary staff, incorporating diverse stakeholder input through mixed-method data collection, including surveys and qualitative sessions. Development involved systematic analysis and validation, ensuring relevance to real-world challenges. Recommendations focus on fostering inclusivity, staff empowerment, shared vision, empathy, ethics, continuous learning, and patient-centered practices, with adaptable implementation strategies. Practical tools and strategies are provided, alongside considerations for resource use, barriers, and evaluation. The process maintained editorial independence, transparency, and plans for regular updates based on stakeholder feedback. These guidelines offer a robust foundation for advancing RL and fostering sustainable leadership improvements in PHC. See Table 2 for final themes shaping RL practices in PHC.

## 3. Discussion and Recommendations

The present study presents the development and evaluation of evidence-based, contextually relevant, and practically applicable RL guidelines designed specifically for PHC settings in South Africa. Developed through a rigorous mixed-method approach integrating quantitative scores from RLQ with qualitative narratives from action learning sessions, reflective journals, and observational data, this guideline addresses systemic leadership challenges within PHC.

Quantitative findings demonstrated low scores in inclusivity, vision, caring, and ethicality consequently suggesting limited application of collaborative and people-centered leadership behaviors. These trends were supported by qualitative insights, where participants described hierarchical decision-making norms, emotional disengagement, and unclear role expectations. These align with prior research indicating that RL in healthcare is often undermined by systemic issues such as staff shortages, managerial workloads, and entrenched command-style leadership cultures [29–31].

The findings of this study present several important considerations for leadership development within PHC organizations. The consistently low scores in visionary leadership highlight a critical deficit in strategic direction-setting, an essential component for organizational cohesion. This gap is echoed in Li et al.'s [31] research, which emphasizes the necessity of a clear organizational vision for fostering team unity and aligning collective efforts. The absence of strong visionary leadership within PHC organizations can result in fragmented decision-making processes and diminished team morale, further underscoring the urgency of addressing this issue.

Moreover, the moderate scores in empowerment, coupled with qualitative reports indicating a desire for greater autonomy among team members, align with Ramamoorthi et al.'s [32] work that links empowerment to enhanced team performance. This suggests that while empowerment practices are somewhat present, they are not robust enough to satisfy the growing need for autonomy in PHC environments. Comparative studies, such as Cougot et al.'s [33] investigation of empowering leadership in healthcare settings, reveal that empowering practices not only reduce stress but also foster innovation and resilience among healthcare teams. This highlights the necessity of improving delegation practices and establishing mechanisms for regular feedback to better support empowerment and autonomy.

Notably, participants highlighted a desire for mentorship, psychological safety, and involvement in shared decision-making. These needs resonate with literature that positions RL as a key determinant of staff engagement, trust, and job satisfaction [34, 35]. Furthermore, the emerging integrated themes such as ethical transparency, empathetic practice, and contextual adaptability are consistent with frameworks advocating for leadership that is reflexive and responsive to the social dynamics of healthcare teams [36, 37].

The guidelines also align with global calls for leadership models that prioritize emotional intelligence, inclusivity, and systems thinking skills that are increasingly critical in navigating healthcare transformation [38, 39]. When effectively implemented, RL can enhance team cohesion, improve care quality, and contribute to resilient organizational cultures [40]. Furthermore, the emphasis on patient-centered leadership in the guidelines responds to growing evidence about the cascading effect of RL on care quality [40]. Furthermore, the inclusion of context-based adaptation mechanisms within the guidelines speaks to [40, 41]'s call for flexible leadership frameworks capable of accommodating the unique challenges of various healthcare environments. By prioritizing relational practices, such as fostering shared leadership and collective problem-solving, the guidelines address critical gaps in traditional hierarchical leadership models, which often fail to leverage team dynamics effectively.

Crucially, this study also highlights the intersection between leadership development and continuous improvement cultures within PHC. The integration of feedback loops, experimental approaches, and reflective practices aligns with [38]'s qualitative study on leadership evolution in complex health systems. However, the findings contribute to filling a gap by demonstrating the importance of embedding RL principles into these cultures to enhance team cohesion and patient outcomes.

The experts found the preliminary guidelines comprehensive and credible but saw a need for clearer refinements. This mirrors challenges in healthcare guidelines development, as noted by [42], balancing academic rigor with practical use. It highlights the need for leadership frameworks that are both theoretically sound and practical in diverse healthcare settings.

While the guidelines were grounded in empirical data and evaluated by experts, it is crucial to note that their effectiveness will depend on sustained organizational commitment, resource availability, and integration into formal leadership development structures. As highlighted by findings in other leadership interventions studies, lack of institutional buy-in, competing priorities, and insufficient mentorship may undermine their impact if not addressed systematically [20, 43].

In conclusion, this study contributes practical, theory-informed, and context-sensitive guidelines for advancing RL capability in PHC. Their strengths are in focusing on daily interactions, ethical conduct, and adaptability, making RL a strategy for cultural change and improving health system responsiveness. By emphasizing relational practices and patient-centered approaches, these guidelines hold the potential to shape the future of leadership in PHC, fostering environments where continuous improvement and collective learning are the norm. Future research should test the guidelines in implementation settings, measure its longitudinal impact on staff and patient outcomes, and explore its scalability across different health service contexts.

## 4. Conclusion

Our results indicate that the RL guidelines for PHC are clinically comprehensive, easy to understand, practical, and valid. These guidelines serve as a standardized tool to guide leaders in fostering inclusivity, empowering teams, and promoting ethical, patient-centered practices within PHC settings. By following this integrated and uniform set of guidelines, inconsistencies and fragmented leadership approaches can be avoided, leading to improved team cohesion, enhanced staff engagement, and better patient outcomes, while ensuring a resilient and adaptive organizational culture. Additionally, the use of these guidelines promotes transparency, continuous learning, and collaboration and reduces ambiguity and variability in leadership practices.

## Figures and Tables

**Table 1:** Demographic and professional attributes of experts: experts panel composition (*N* = 10).

Attribute	Descriptor	Frequency
Gender	Male	4 (40%)
	Female	6 (60%)

Age	34–39 years	1 (10%)
	40–50 years	3 (30%)
	Above 50 years	6 (60%)

Employment position	Lecturer—nursing education	2 (20%)
	Healthcare leaders	5 (50%)
	Specialist nurse—mental health	1 (10%)
	Professor—nursing	2 (20%)

Area of expertise	Healthcare leadership	3 (30%)
	Relational care/RL	4 (40%)
	Guidelines development	3 (30%)

Years of experience in area of expertise	5–10 years	4 (40%)
	11+ years	6 (60%)

Highest academic qualification	Master's degree	4 (40%)
	Doctoral degree	6 (60%)

**Table 2:** Key themes and components for RL practices in PHC.

Theme	Key components
Fostering inclusivity and participation	Collaborative decision-making, psychological safety
Enhancing empowerment through mentorship	Delegation strategies, leadership development
Developing and communicating a shared vision	Clear organizational direction, forward-thinking leadership
Exhibiting caring and empathy	Emotional intelligence, relational awareness
Upholding ethical leadership	Transparent decision-making, integrity in leadership
Promoting continuous learning	Reflexive leadership, action-learning cycles
Integrating patient-centered leadership	Role modeling patient-centered care, staff engagement
Context-based leadership with feedback	Adaptive interventions, stakeholder collaboration

## Data Availability

The data that support the findings of this study are available from the corresponding author upon reasonable request.

## Nomenclature

AGREE II: Appraisal of guidelines for research and evaluation II
PHC: Primary healthcare
RL: Relational leadership
RLQ: Relational leadership questionnaire
RLT: Relational leadership theory
